# The Significance of MAPK Signaling Pathway in the Diagnosis and Subtype Classification of Intervertebral Disc Degeneration

**DOI:** 10.1002/jsp2.70060

**Published:** 2025-03-24

**Authors:** Yong Liu, Xueyan Chen, Jingwen Chen, Chao Song, Zhangchao Wei, Zongchao Liu, Fei Liu

**Affiliations:** ^1^ Department of Orthopedics, The Affiliated Hospital of Traditional Chinese Medicine Southwest Medical University Luzhou China; ^2^ Department of Anesthesiology, The Affiliated Traditional Chinese Medicine Hospital Southwest Medical University Luzhou China; ^3^ Department of Orthopedics Luzhou Longmatan District People's Hospital Luzhou China; ^4^ Department of Orthopedics RuiKang Hospital Affiliated to Guangxi University of Chinese Medicine Nanning China

**Keywords:** consensus clustering, immune cell infiltration, intervertebral disc degeneration, MAPK signaling pathway

## Abstract

**Background:**

Intervertebral disc degeneration (IDD) is a human aging disease related mainly to inflammation, cellular senescence, RNA/DNA methylation, and ECM. The mitogen‐activated protein kinase (MAPK) signaling pathway is engaged in multiple biological functions by phosphorylating specific serine and threonine residues on target proteins through phosphorylation cascade effects, but the role and specific mechanisms of the MAPK signaling pathway in IDD are still unclear.

**Methods:**

We identified 20 MAPK‐related differential genes by differential analysis of the GSE124272 and GSE150408 datasets from the GEO database. To explore the biological functions of these differential genes in humans, we performed GO and KEGG analyses. Additionally, we applied PPI networks, LASSO analysis, the RF algorithm, and the SVM‐RFE algorithm to identify core MAPK‐related genes. Finally, we conducted further validation using clinical samples.

**Results:**

We ultimately identified and validated four pivotal MAPK‐related genes, namely, KRAS, JUN, RAP1B, and TNF, using clinical samples, and constructed the ROC curves to evaluate the predictive accuracy of the hub genes. A nomogram model was subsequently developed based on these four hub MAPK genes to predict the prevalence of IDD. Based on these four hub genes, we classified IDD patients into two MAP clusters by applying the consensus clustering method and identified 1916 DEGs by analyzing the differences between the two clusters. Further analysis using the same approach allowed us to identify two gene clusters based on these DEGs. We used a PCA algorithm to determine the MAPK score for each sample and discovered that MAPK cluster A and gene cluster A had higher scores, suggesting greater sensitivity to MAPK signaling pathway‐associated agents in the subtype. We displayed the differing expression levels of four hub MAPK‐related genes across the two clusters and their relationship with immune cell infiltration to highlight the distinctions between clusters A and B.

**Conclusion:**

In summary, four hub MAPK signaling pathway‐related genes, KRAS, JUN, RAP1B, and TNF, could be applied to the diagnosis and subtype classification of IDD and benefit the prevention and treatment of IDD.

## Graphical abstract

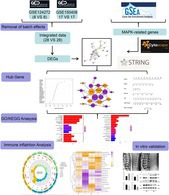

## 1 Introduction

The intervertebral discs (IDs) are cartilaginous tissues between individual spinal segments that help maintain flexibility between vertebrae and transmit compressive loads to each segment [1]. Intervertebral disc degeneration (IDD) is a degenerative disease of the musculoskeletal system that occurs with advancing age [2]. IDD not only diminishes the overall quality of life for affected individuals but also imposes substantial economic burdens on society as a whole. The current majority view is that IDD primarily arose from cellular aging, extracellular matrix (ECM) breakdown, and oxidative stress [3]. The current clinical treatment of IDD can be broadly classified as conservative treatment followed by surgery. Conservative treatment mainly includes oral pain medication, functional exercise, acupuncture, and massage, as well as promising therapies such as gene therapy, molecular therapy, and biologics that are being studied [4]. Surgical treatment includes spinal decompression, lumbar fusion, and discectomy. However, none of the above‐mentioned treatments can reverse the biomechanical changes in the entire spine caused by disc degeneration and are mainly aimed at relieving pain [5, 6]. Therefore, early diagnosis and identification of different subtype classifications are of great significance in guiding clinical treatment plans.

ZxMitogen‐activated protein kinases (MAPKs) are a conserved cohort of intracellular signaling proteins that assume a crucial function in the regulation of diverse cellular mechanisms. MAPKs are activated in response to multiple extracellular and intracellular stimuli by a highly complex and tightly regulated signaling cascade involving upstream signaling molecules [5, 6]. MAPKs play a critical role in the activation, maturation, and differentiation of immune cells. Upon activation, MAPKs phosphorylate downstream targets, including transcription factors, enabling them to modulate gene expression and regulate various steps in immune cell development, activation, and effector function [7]. Notably, the various biological functions of MAPK kinases need to be activated through a complex three‐layer kinase cascade reaction, i.e., MAPK kinase initiates the activation of MAPK kinase, subsequently leading to the activation of the respective targeted MAPK. Nevertheless, malfunctions in the functional dynamics of this intricate cascade mechanism play a contributory role in the etiology of diverse pathological conditions [8]. Yang et al. discovered that Eupatilin, a flavonoid isolated from *Artemisia annua* , possesses the ability to impede extracellular matrix (ECM) degradation and ameliorate senescence in nucleus pulposus (NP) cells by suppressing TNF‐α‐induced MAPK/NF‐κB activation, ultimately exerting a remarkable effect in delaying the progression of IDD [9]. Dai et al.'s investigation suggests that STS could potentially mitigate IDD and oxidative stress in rats by inhibiting p38MAPK activity [10]. Furthermore, compounds such as wogonin, quercetin, and BRD4 have demonstrated the capacity to alleviate disc degeneration by modulating the MAPK signaling pathway [11, 12]. The activation of the MAPK signaling pathway is known to stimulate the degradation of the extracellular matrix of IDs, as well as the onset of cellular senescence, apoptosis, and inflammatory reactions. Additionally, it triggers autophagic and oxidative stress responses, which hasten the degenerative processes of the IDs [13]. Despite the fact that the study of the MAPK signaling pathway in IDD has gained popularity and that more researchers have attempted to create drugs that target the MAPK signaling pathway for the treatment of IDD, regrettably no satisfactory results have been obtained, and the precise role of the MAPK signaling pathway in IDD is still not fully understood.

This study uniquely integrates advanced bioinformatics analyses to explore the diagnostic and therapeutic potential of MAPK‐related genes in IDD, providing a novel perspective on subtype classification. By examining the GSE124272 and GSE150408 datasets, we thoroughly assessed the contribution of genes related to the MAPK signaling pathway to the diagnosis and subtype classification of IDD in our study. We first obtained four core MAPK signaling pathway‐related genes (KRAS, JUN, RAP1B, and TNF), based on which we built a nomogram to predict the prevalence of IDD [14, 15]. Furthermore, we have delineated two discrete MAPK or gene clusters that exhibit consistent patterns of differential gene expression associated with the onset of IDD. Finally, we further validated the expression levels of these genes using clinical samples. These findings suggest that our established model holds potential for risk evaluation and categorization of IDD subtypes. It will also serve as a guide for future research on MAPK signaling pathways in IDD and the creation of clinical treatment approaches for IDD.

## 2 Materials and Methods

### 2.1 Data Preprocessing, Integration, and Differential Expression Analysis of MAPK‐Related Genes in IDD

The GEO database hosts two data sets, namely GSE124272 and GSE150408, comprising 25 healthy and 25 IDD samples [16, 17]. The GSE124272 data set encompasses 8 healthy and 8 IDD samples, whereas the GSE150408 data set comprises 17 healthy and 17 IDD samples. These data sets are based on the GPL21185 annotation platform. Initially, we performed log2 transformation of the data sets and separately normalized the resulting expression values. Subsequently, we integrated these data sets and employed the “ComBat” function to mitigate the batch effect [18, 19]. This adjustment ensures that any systematic differences introduced by technical variability in sample processing are minimized, allowing for a more robust and accurate analysis of the integrated data set. We obtained a list of MAPK‐related genes from the gene set enrichment analysis (GSEA) database [20] (Table S1). To assess their potential involvement in IDD, we performed differential expression analysis (*p* < 0.01) between healthy and IDD samples, identifying a set of MAPK‐related genes that were differentially expressed [20, 21]. To further investigate the biological pathways and processes in which these differentially expressed MAPK‐related genes may be involved, we conducted Gene Ontology (GO) and Kyoto Encyclopedia of Genes and Genomes (KEGG) analyses (*p* < 0.05) [22]. Our analysis resulted in the identification of several significantly enriched GO and KEGG pathways associated with MAPK‐related genes in IDD.

### 2.2 Identification and Validation of Hub MAPK‐Related Genes in IDD

To identify the hub MAPK‐related genes involved in IDD, we employed a sophisticated analytical approach that consisted of several computational tools and algorithms. The protein–protein interaction (PPI) network (interaction score > = 0.4) [23], least absolute shrinkage and selection operator (LASSO) analysis (10‐fold cross‐validation), random forest (RF) algorithm (ntree = 500), and support vector machine recursive feature elimination (SVM‐RFE) algorithm were used to identify hub MAPK‐related genes [24]. This method utilizes a regularization technique that shrinks coefficients of unimportant features to zero, thereby enhancing the predictive power of the model. We also employed a random forest (RF) algorithm with ntree set at 500 to identify the key genes relevant to IDD pathogenesis. The RF algorithm is a machine learning method that utilizes an ensemble of decision trees to predict target variables and identify important features. The reasons for selecting the RF algorithm included the ability to perform feature importance assessment, excellent robustness and noise resistance, and no need for normalization. Lastly, we used the support vector machine recursive feature elimination (SVM‐RFE) algorithm to rank genes based on their relevance to IDD [25]. This algorithm eliminates redundant and irrelevant features iteratively, leading to a subset of informative genes. The reasons for selecting the SVM‐RFE algorithm included being adept at handling small sample data, distinguishing features with high impact, and having excellent generalization ability. By integrating the results from these computational methods, we identified a set of hub MAPK‐related genes that are highly likely involved in IDD pathogenesis. Finally, a set of central MAPK‐related genes was identified through the integration of four analytical techniques. A nomogram is a graphical statistical tool used to predict the probability of an event occurring or the risk of developing a disease. These genes were employed to develop a nomogram for predicting the incidence of IDD. The Receiver Operating Characteristic (ROC) curve and Area Under ROC Curve (AUC) value were employed to gauge the precision and reliability of the hub MAPK‐related genes [26].

### 2.3 Identification and Characterization of MAPK‐Related Molecular Classification

The R package “ConsensusClusterPlus” was applied to recognize different MAPK clusters based on MAPK‐related genes [27]. Next, employing principal component analysis (PCA), we scrutinized the expression of MAPK‐associated genes and deduced that diverse MAPK clusters were correlated with immune cell infiltration. We selected differentially expressed genes (DEGs) from distinct MAPK clusters and availed ourselves of analogous procedures as delineated earlier to derive distinct gene clusters (*p* < 0.05 and |log2FC| > 0.585) [28].

### 2.4 Evaluating Gene Expression and MAPK Activity in IDD

In this study, the MAPK score was utilized to evaluate the expression of genes associated with MAPK signaling in samples from individuals with IDD. This scoring system was calculated using the sum of principal components 1 through 5 for each gene i, resulting in the formula: Σ(PC1i + PC2i + PC3i + PC4i + PC5i) = MAPK score. This approach offers a comprehensive analysis of multiple genes and their overall impact on MAPK activity in IDD. In addition, the expression levels of genes that have been confirmed to be associated with the development of IDD were also assessed in different clusters [29].

### 2.5 Collection of Intervertebral Disc Tissue Samples

Intervertebral disc tissues were obtained for this study with informed consent from the patients' families and approval from the Ethics Committee of the Affiliated Traditional Chinese Medicine Hospital of Southwest Medical University (Ethics Approval Number: KY2022062‐FS01). Samples were collected from patients with IDD and those requiring surgery for lumbar burst fracture (LBF). (1) IDD Group: IDD specimens were collected from the protrusion sites of 4 patients with IDD. These patients were classified as Grade 4 or 5 according to the Pfirrmann grading system. (2) LBF Group: FNF samples were obtained from the non‐injured regions of 4 patients with LBF. The exclusion criteria for both groups included: (1) coexisting bone diseases such as osteoarthritis, Paget's disease, or bone tumors; (2) pre‐existing conditions prior to the study, such as cancer, cardiovascular disease, or diabetes; (3) patients who did not consent to the study protocol. All samples were promptly stored in liquid nitrogen until use. The clinical characteristics of the patients are summarized in Supplementary Table S6.

### 2.6 Real‐Time PCR Analysis

The gene primer sequences were designed and synthesized by Sangon Biotech (Shanghai, China). The primer sequences for each gene are listed in Table 1. Total RNA was extracted using a bone tissue RNA extraction kit (Aidlab, #160902) after tissue homogenization. Complementary DNA (cDNA) was synthesized using the PrimeScript RT reagent kit with gDNA Eraser (Invitrogen, #AM2071). qRT‐PCR was performed using the SYBR Green qPCR Master Mix (Applied Biosystems, #A46012) on the 7500 Real‐Time PCR System (Thermo Fisher Scientific, Waltham, MA, USA) with a total of 40 cycles. GAPDH was used as the internal control, and the relative expression levels of each gene were calculated using the 2^−ΔΔCT^ method.

**TABLE 1:** The primer sequences used for PCR amplifcation.

Gene	Forward primer seguence	Revere primer sequence
JUN	GAGCGGACCTTATGGCTACA	CCCGTTGCTGGACTGGATT
KRAS	TGGGGAGGGCTTTCTTTGTGTA	GGACCATAGGTACATCTTCA
RAP1B	CTGGGAAGGCTCGCAAA	CCACCACAGGAAAGTCCATT
TNF	TGGGCAGGTCTACTTTGGGA	GAGGTTGAGGGTGTCTGAAG
GAPDH	GCACCGTCAAGGCTGAGAAC	TGGTGAAGACGCCAGTGGA

### 2.7 Western Blot

Intervertebral disc tissues from each group were rinsed three times with cold phosphate‐buffered saline (PBS) and then ground in liquid nitrogen. Total protein was extracted using RIPA lysis buffer (Solarbio, R0010). Total protein concentration was measured using a BCA protein assay kit (Solarbio, PC0020). Equal amounts of protein from each sample were separated by SDS‐PAGE and then transferred to a polyvinylidene difluoride (PVDF) membrane. The membranes were blocked with 5% non‐fat milk at room temperature for 2 h, followed by overnight incubation with the primary antibody at 4°C. The PVDF membranes were then incubated with the secondary antibody at room temperature for 2 h. GAPDH was used as the loading control. Densitometric analysis of the images was performed using ImageJ software version 1.8.

### 2.8 Statistical Analysis

Statistical analyses and graphing were performed using GraphPad Prism 9.0 software. Data are presented as mean ± standard error of the mean (SEM) from three or more independent experiments. For two‐group comparisons, a t‐test was used if the data were normally distributed; otherwise, a nonparametric test was applied. One‐way analysis of variance (ANOVA) was used to compare samples from different groups. Post hoc comparisons between groups were conducted using Tukey's test. Statistical significance was denoted by *p < 0.05, and highly significant differences were denoted by **p < 0.01.

## 3 Results

### 3.1 Landscape of MAPK‐Related Genes

**FIGURE 1:**
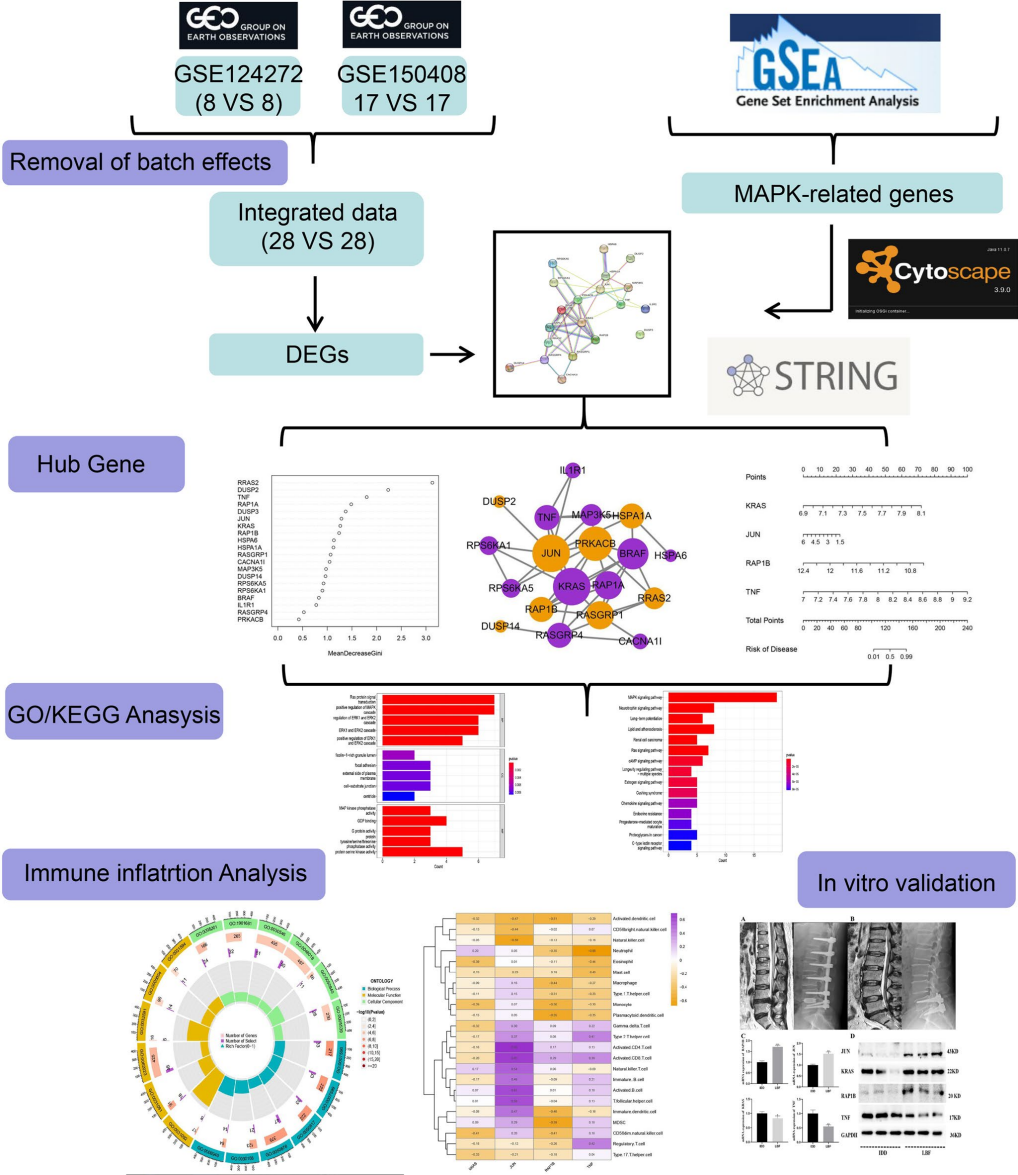
Article Flowchart.

**FIGURE 2:**
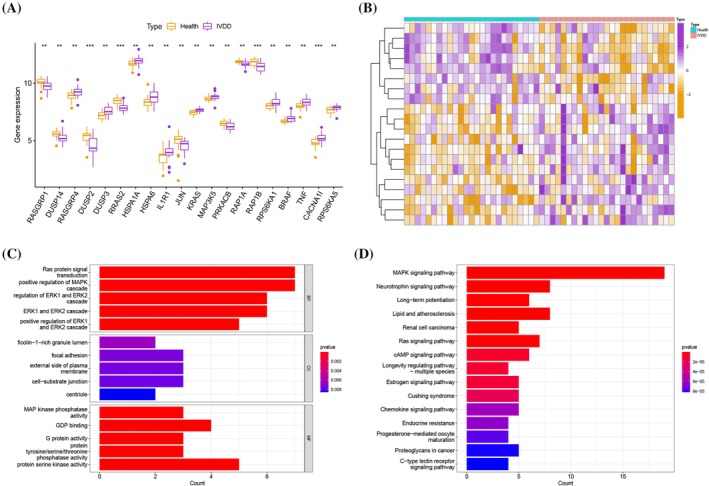
(A, B) 20 differentially expressed MAPK‐related genes. (C) GO enrichment analysis for 20 differentially expressed MAPK‐related genes. (D) KEGG enrichment analysis for 20 differentially expressed MAPK‐related genes.

The bioinformatics analysis workflow of this study is shown in Figure 1. We obtained 20 differentially expressed MAPK‐related genes, of which RASGRP4, DUSP3, HSPA1A, HSPA6, IL1R1, KRAS, MAP3K5, RPS6KA1, BRAF, TNF, CACNA1I, and RPS6KA5 were overexpressed in IDD samples, whereas RASGRP1, DUSP14, DUSP2, RRAS2, JUN, PRKACB, RAP1A, and RAP1B were downregulated (Figure 2A,B). We researched the functions and molecular pathways of 20 differentially expressed MAPK‐related genes in IDD. Biological process (BP) terms are related to Ras protein signal transduction, positive regulation of MAPK cascades, and regulation of ERK1 and ERK2 cascades; cellular component (CC) terms are related to ficolin‐1‐rich granule lumen, focal adhesion, and external side of plasma membrane; and molecular function (MF) terms are related to MAP kinase phosphatase activity, GDP binding, and G protein activity (Figure 2C Supplementary Table S2). KEGG terms are enriched mainly in MAPK signaling pathway, neurotrophin signaling pathway, long‐term potentiation, lipid and atherosclerosis, renal cell carcinoma, and Ras signaling pathway (Figure 2D and Supplementary Table S3).

### 3.2 Identification of Hub MAPK‐Related Genes

**FIGURE 3:**
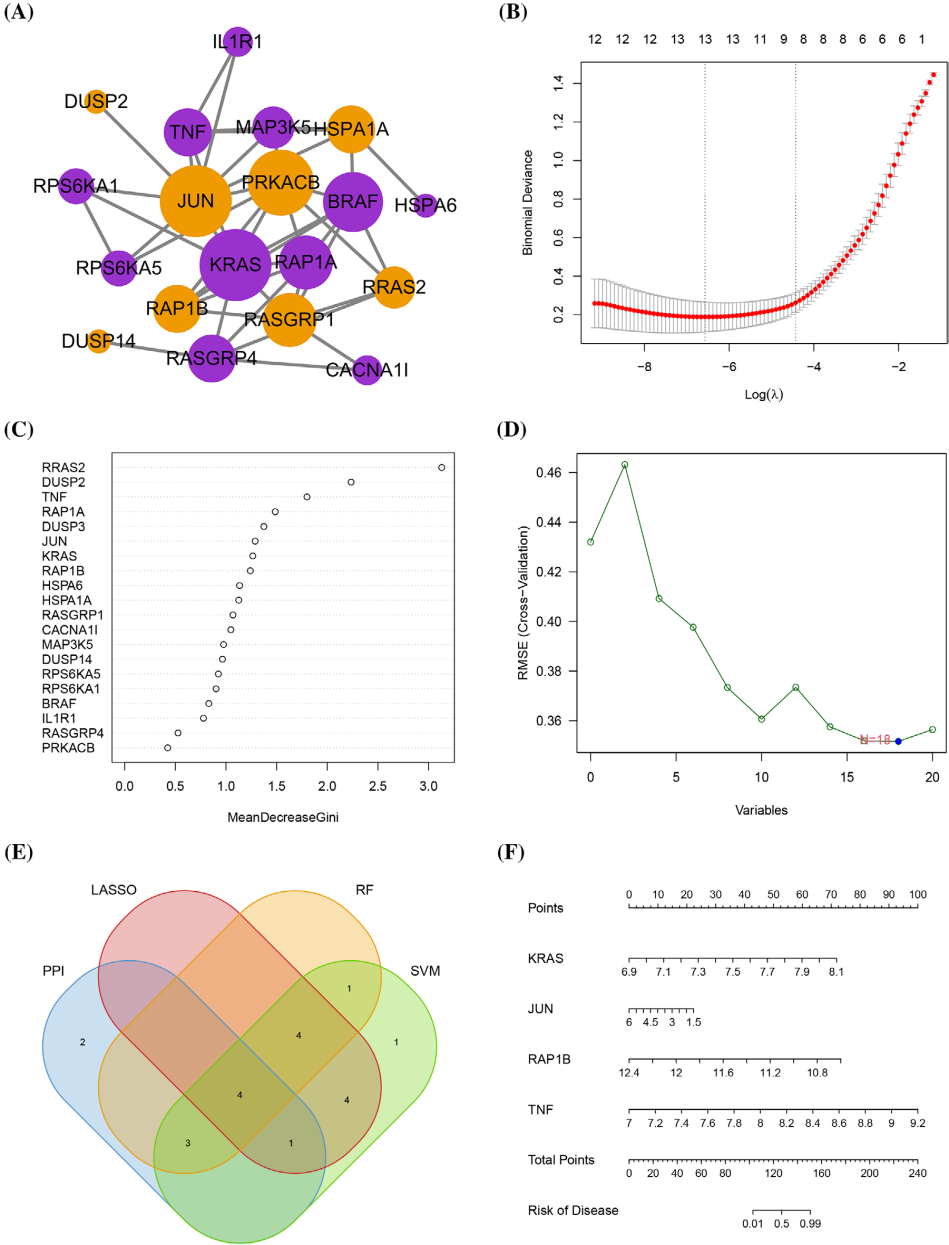
(A) The PPI network. (B) 13 key MAPK‐related genes by the LASSO algorithm. (C) 12 key MAPK‐related genes by the RF algorithm. (D) 18 key MAPK‐related genes by the SVM‐RFE algorithm. (E) Four hub MAPK‐related genes (KRAS, JUN, RAP1B, and TNF). (F) A nomogram based on these four hub MAPK‐related genes.

We identified 10 key MAPK‐related genes by the PPI network (Figure 3A and Figure S1), 13 key MAPK‐related genes by the LASSO algorithm (Figure 3B), 12 key MAPK‐related genes by the RF algorithm (Figure 3C), and 18 key MAPK‐related genes by the SVM‐RFE algorithm (Figure 3D). Then, we intersected the results of the four different analyses mentioned above to acquire four hub MAPK‐related genes (KRAS, JUN, RAP1B, and TNF) that were significantly related to IDD (Figure 3E). Finally, we constructed a nomogram to predict the incidence of IDD based on these four hub MAPK‐related genes (Figure 3F). The AUC values of JUN, KRAS, RAP1B, and TNF were 0.728, 0.725, 0.776, and 0.737, respectively, indicating that the four hub MAPK‐related genes had a high accuracy and predictive value (Figure S2).

### 3.3 Identification and Characterization of MAPK‐Related Molecular Classification

**FIGURE 4:**
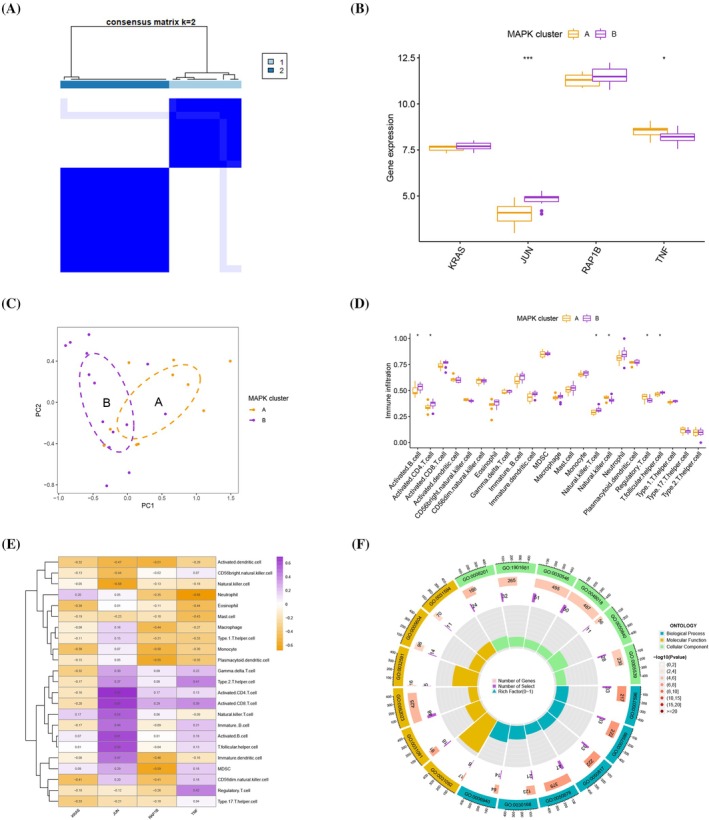
(A) Consensus matrices of four hub MAPK‐related genes for k = 2. (B) The expression levels of JUN and TNF were statistically different in different MAPK clusters. (C) PCA indicated that the two MAPK clusters could be discriminated. (D) Differences in immune cell infiltration analysis between different MAPK clusters. (E) The relationship between individual hub MAPK‐related genes and immune cell infiltration. (F) GO enrichment analysis for 1916 DEGs.

Two MAPK clusters (MAPK clusters A and B) were identified by using the consensus clustering method based on the four hub MAPK‐related genes (Figure 4A and Figure S3). The expression levels of KRAS and RAP1B in different MAPK clusters were not statistically different, whereas the differences in the expression levels of JUN and TNF in different MAPK clusters were statistically significant (Figure 4B). PCA indicated that the two MAPK clusters could be discriminated (Figure 4C). Immune cell infiltration analysis indicated that natural killer cells and regulatory T cells are enriched in MAPK cluster A, while activated B cells, activated CD4 T cells, natural killer T cells, and T follicular helper cells are enriched in MAPK cluster B (Figure 4D). Moreover, we explored the relationship between individual hub MAPK‐related genes and immune cell infiltration (Figure 4E). We identified 1916 DEGs between different MAPK clusters (*p* < 0.05 and |log2FC| > 0.585) (Supplementary Table S4). The GO analysis indicated that BP terms are related to blood coagulation, hemostasis, and coagulation; CC terms are related to platelet alpha granule membrane, platelet alpha granules, and collagen‐containing extracellular matrix; and MF terms are related to heparin binding, sulfur compound binding, and signaling receptor activator activity (Figure 4F and Supplementary Table S5).

### 3.4 Identification and Characterization of Gene Molecular Classification

**FIGURE 5:**
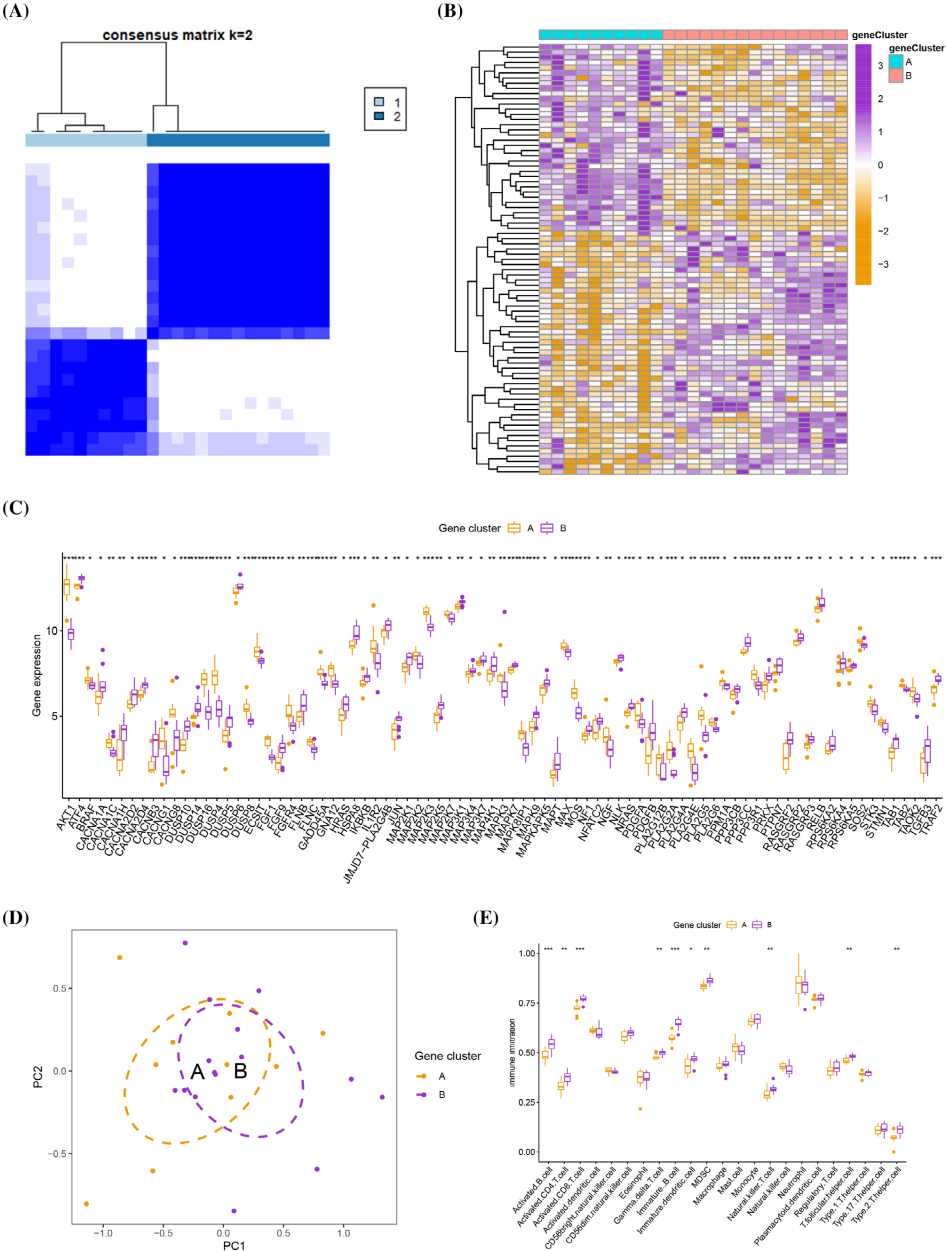
(A) Consensus matrices of 1916 DEGs for k = 2. (B, C) The expression levels of various MAPK‐related genes were statistically different between different gene clusters. (D) PCA indicated that the two gene clusters could be discriminated. (E) Differences in immune cell infiltration analysis between different gene clusters.

Two gene clusters (gene clusters A and B) were identified by using the consensus clustering method based on the 1916 DEGs (Figure 5A and Figure S4). The expression levels of various MAPK‐related genes were statistically different between the different gene clusters (Figure 5B,C). PCA indicated that the two gene clusters could be discriminated (Figure 5D). Immune cell infiltration analysis indicated that activated B cells, activated CD4 T cells, activated CD8 T cells, gamma delta T cells, immature B cells, immature dendritic cells, myeloid‐derived suppressor cells (MDSC), natural killer T cells, T follicular helper cells, and type 2 T helper cells are enriched in gene cluster B (Figure 5E).

### 3.5 Evaluating Gene Expression and MAPK Activity in IDD

**FIGURE 6:**
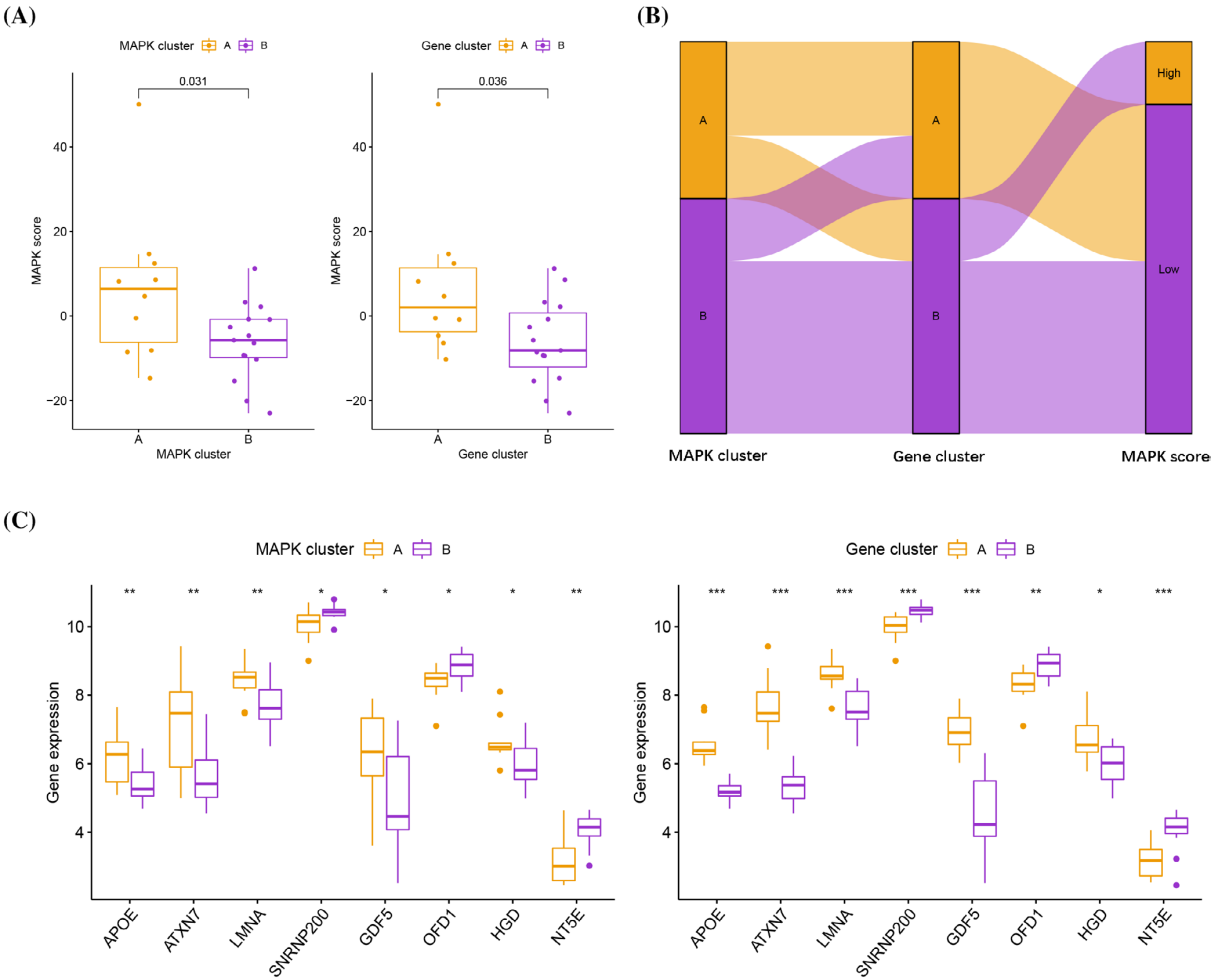
(A) MAPK scores of MAPK cluster A or gene cluster A were significantly higher than those of MAPK cluster B or gene cluster B. (B) The Sankey plot shows the relationship between MAPK clusters, gene clusters, and MAPK scores. (C) The expression levels of genes that have been confirmed to be related to the development of IDD were assessed in different clusters.

We employed the PCA algorithm to compare MAPK scores between different MAPK clusters or gene clusters. MAPK scores for MAPK cluster A or gene cluster A were significantly higher than MAPK cluster B or gene cluster B, suggesting greater sensitivity to MAPK‐associated agents in the subtype (Figure 6A). The Sankey plot shows the relationship between MAPK clusters, gene clusters, and MAPK scores (Figure 6B). The expression levels of genes that have been confirmed to be associated with the development of IDD were assessed in different clusters, of which APOE, ATXN7, LMNA, GDF5, and HGD were overexpressed in MAPK cluster A and gene cluster A, whereas SNRNP200, OFD1, and NT5E were downregulated in MAPK cluster A and gene cluster A (Figure 6C).

### 3.6 Validation of Core Genes

**FIGURE 7:**
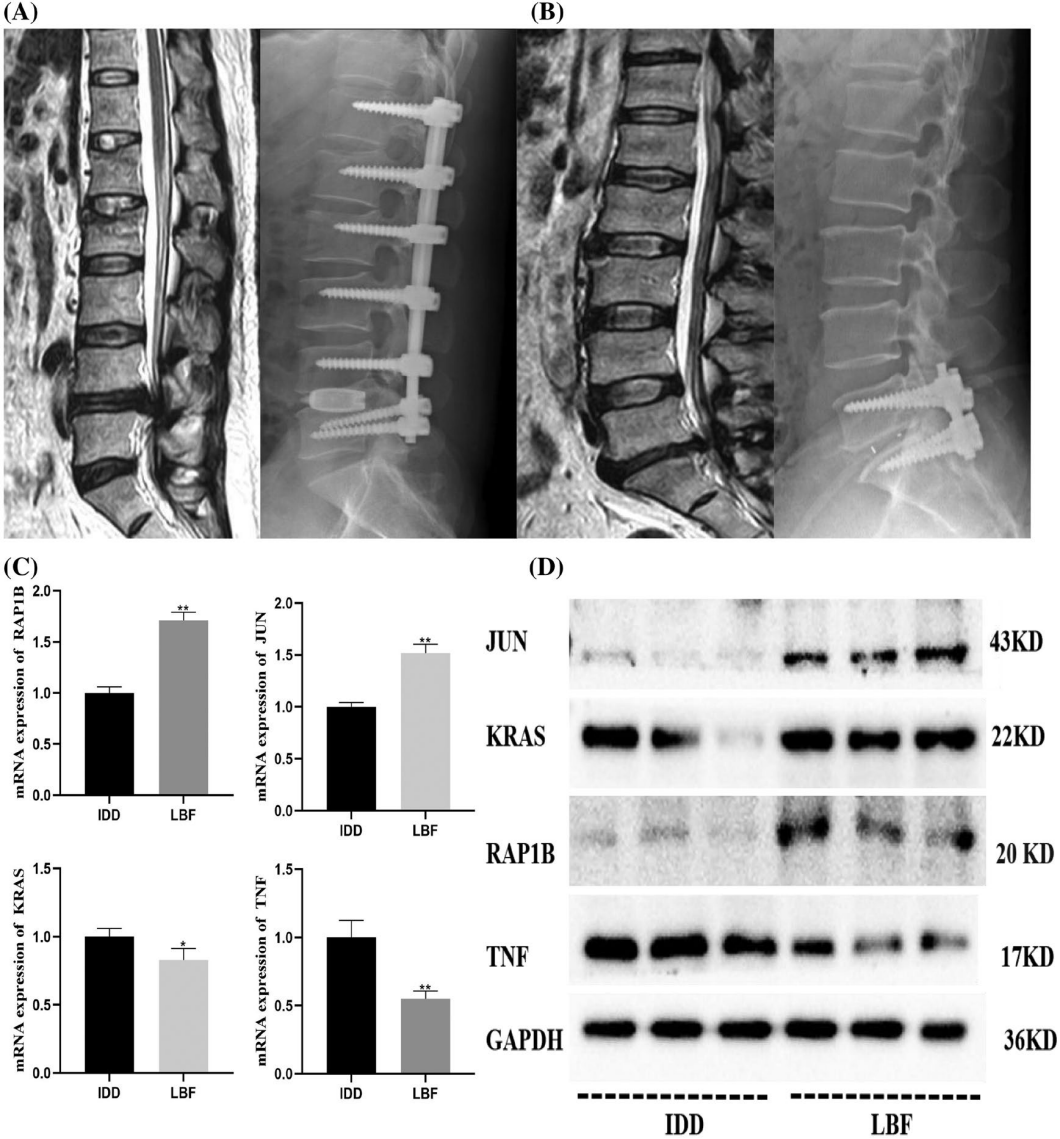
Differences in key gene protein expression between IDD and LBF tissues. (A, B) Preoperative MRI and postoperative X‐ray images of LBF and IDD patients. (C) mRNA expression of key genes (*n* = 5, JUN **p < 0.01 vs. LBF, RAP1B **p < 0.01 vs. LBF, KRAS *p < 0.05 vs. LBF, TNF **p < 0.01 vs. LBF; two‐group comparisons were performed using t‐tests). (D) Protein expression of key genes (*n* = 3).

Through bioinformatics data analysis, we identified four key genes related to MAPK expression (JUN, RAP1B, KRAS, TNF). Based on these results, we collected clinical tissue samples from 5 IDD patients and 5 LBF patients (Figure 7A,B) to further validate the identified core genes. The qRT‐PCR results showed that, compared to LBF, JUN and RAP1B expression were decreased, while KRAS and TNF expression were increased in IDD (Figure 7C). Western blot analysis revealed a similar trend, with decreased JUN and RAP1B expression and increased KRAS and TNF expression in IDD compared to LBF (Figure 7D) (*p* < 0.05). In summary, four hub MAPK signaling pathway‐related genes, KRAS, JUN, RAP1B, and TNF, could be applied to the diagnosis and subtype classification of IDD and benefit the prevention and treatment of IDD.

## 4 DISCUSSION

According to statistics, more than 84% of people worldwide experience low back pain, and IDD is a main pathological factor in low back pain, but its pathological mechanism is still not fully elucidated [30]. The ID is the cartilaginous tissue seen adjacent to the vertebral body and consists of chondrocyte‐like annulus fibrosus (AF) tissue, colloid NP tissue, and cartilage end plate (CEP) tissue [31]. The reticular degeneration and hyalinization of fibrous ring tissue, water loss in the gelatinous medullary tissue, higher levels of immune cell infiltration, and inflammatory mediators all contribute to the development of IDD, a chronic inflammatory process that inevitably leads to disc degeneration [32, 33]. Notably, the activation of MAPK is presumed to stimulate ECM catabolism, instigate inflammatory reactions, induce cellular senescence and apoptotic events, as well as expedite disease advancement in IDD by triggering oxidative stress and autophagy [13]. Regrettably, the precise function of the MAPK signaling pathway and its underlying mechanism of operation within the context of IDD remain incompletely understood.

This study represents the first attempt to investigate the role of the MAPK signaling pathway in the progression of IDD through a combination of bioinformatics analysis and validation with clinical samples. In the research, we identified 20 differentially expressed MAPK‐related genes. We then identified four hub MAPK‐related genes, namely KRAS, JUN, RAP1B, and TNF, by combining the PPI network, LASSO analysis, SVM‐RFE algorithm, and RF algorithm. We validated the expression of the aforementioned genes in tissue samples from IDD and lumbar disc herniation (LBF), finding that the expression of JUN and RAP1B was reduced in IDD compared to LBF, while KRAS and TNF expression was increased. KRAS, one of the three RAS genes, can be selectively spliced to produce approximately 12 kDa‐long KRAS4A and KRAS4B with nearly identical G structural domains and is one of the common genetic mutations in many human cancers, playing a central role in cancer biology [34, 35, 36]. PPDPF, which is highly expressed in pancreatic cancer, regulates the GEF activity of SOS1 to promote disease progression in Kras mutant pancreatic ductal carcinoma [37]. Originally named for its ability to induce hemorrhagic tumor necrosis, TNF, produced mainly by macrophages and T lymphocytes is a key cytokine linking the body's immune system and inflammatory response and is highly related to the development of IDD [38, 39]. Liu et al. showed that Fexofenadine could inhibit TNF‐α‐mediated ECM degradation and disc degeneration by modulating the cPLA2/NF‐κB signaling pathway [40]. Wang et al. found that downregulation of Follistatin‐Like 1 significantly attenuated the TNF‐α‐induced inflammatory response and reduced the expression levels of inflammatory factors such as iNOS, COX‐2, and MMP‐13 in IDD, ultimately delaying the progression of IDD [41]. AP‐1, also known as transcription factor Jun, is considered a key transcription factor in tumorigenesis and is composed of Jun‐ (JUNB, JUND, Jun), ATF‐ (ATF‐2, ATF‐3/LRF1, ATF‐4, etc.), Fos‐ (FRA1, FRA2, Fos, FOSB), and MAF (c‐MAF, MAFA, MAFB, etc.) homodimers or heterodimers [42]. AP‐1 is highly expressed in various tumors and exerts multiple biological functions, including promoting proliferation, migration, invasion, apoptosis, and angiogenesis [43]. Yu et al. discovered that the herbal monomer ailanthone could function as a c‐Jun inhibitor by exerting pro‐c‐Jun degradation, directly inhibiting c‐Jun‐induced PD‐L1 expression and secretion, and lowering the level of treg cell infiltration in the tumor microenvironment, improving the therapeutic effect of anti‐pd‐L1 on melanoma [44]. RAP1B is a member of the Ras family of small G proteins and shares 95% homology with another isoform member, Rap1a, and has been shown to inhibit angiogenesis, block MAPK signaling pathway activation, and impair endothelial cell migration and proliferation [45]. Rap1b plays a role in pro‐proliferation, invasion, migration, and EMT in a variety of malignancies [46].

These hub genes (KRAS, JUN, RAP1B, and TNF) can be used in the diagnosis or treatment of IDD in several impactful ways. By detecting the expression levels of KRAS, JUN, RAP1B, and TNF genes, doctors can recognize the occurrence of IDD earlier and assess the degree of disease progression, as shown in the nomogram graph, where high expression of KRAS and TNF, and low expression of JUN and RAP1B, are associated with having IDD. The expression profiles of these genes can also guide doctors in identifying molecular subtypes and personalized treatment for IDD patients. For example, IDD patients with MAPK cluster A or gene cluster A are more likely to benefit from drugs related to the MAPK signaling pathway. Although all four hub MAPK‐related genes are known to play a role in various tumors, their roles and mechanisms in disc degeneration remain incompletely understood, especially KRAS, JUN, and RAP1B, which have been less studied. Therefore, we expect that our research will provide guidance for future inquiries into the contribution of these four central MAPK‐related genes to IDD.

It is currently well established that the onset and progression of IDD are significantly influenced by the immune system's response and inflammation [47]. Nonetheless, it is also well recognized that the NP, under standard physiological conditions, represents the largest immune‐privileged organ within the body, largely due to its distinctive location within a specialized enclosed space that is enveloped by the outer layer of the annulus fibrosus and the upper and lower regions of the cartilaginous endplate [48]. However, in the event of a rupture in the fibrous immune barrier known as the fibrous immune barrier, the study of philosophy leads to a significant influx of immune cells. This leads to a heightened presence of these immune cells within the body [49]. Nonetheless, the immune cells that infiltrate the affected area actively secrete substantial quantities of inflammatory cytokines, thereby intensifying the cascade of inflammatory responses and ECM degradation. Additionally, these cytokines facilitate the progression of IDD [49, 50]. Employing the consensus clustering algorithm, we successfully identified two distinct clusters related to the MAPK pathway, namely MAPK cluster A and MAPK cluster B. These clusters were determined based on the expression profiles of four pivotal MAPK‐related genes, namely JUN, RAP1B, TNF, and KRAS. Additionally, we employed the PCA algorithm to compute the MAPK score for each individual sample, which revealed that MAPK cluster A or gene cluster A exhibited a higher score compared to MAPK cluster B or gene cluster B, suggesting greater sensitivity to MAPK signaling pathway‐associated agents in the subtype. This suggests that the MAPK signaling pathway is more actively expressed in MAPK cluster A. Further investigation revealed that MAPK cluster B had higher immune cell infiltration levels, including activated B cells, CD4 T cells, natural killer cells, and T follicular helper cells. It appears that MAPK cluster B is linked to the onset and progression of IDD. We analyzed the expression differences of IDD‐related genes between the two clusters; notably, APOE and GDF5 were expressed at higher levels in MAPK cluster A, and we know that both APOE and GDF5 function to maintain normal physiological structure and function of the ID and inhibit disc degeneration [51, 52]. Finally, to quantify the MAPK cluster, we calculate the MAPK score of each sample by applying the PCA algorithm and find that cluster B has a lower score compared to cluster A.

However, it is important to acknowledge several limitations in this study. First, the absence of clinical data in the utilized data sets prevents further exploration of the association between clinical information and different subtypes. Second, the small sample size employed in this study may introduce potential bias. Larger sample sizes are needed to ensure the robustness and generalizability of the findings, as small sample sizes can result in skewed results and limited statistical power. In light of these limitations, future research endeavors should focus on collecting a larger number of clinical samples, recording more comprehensive clinical information, and conducting experiments, as well as clinical cohort studies, to further examine and validate the outcomes reported in this study.

## 5 Conclusion

In brief, we have developed a prognostic nomogram model that utilizes four genes (KRAS, JUN, RAP1B, and TNF) that are implicated in the hub MAPK signaling pathway to predict the prevalence of IDD patients. Additionally, we validated the expression levels of these genes in clinical samples. By utilizing consensus clustering, we have constructed two distinct MAPK clusters (A and B), wherein cluster B exhibits a higher degree of immune cell infiltration and differential expression patterns of IDD‐related genes as compared with cluster A, thereby suggesting an association between MAPK cluster B and IDD. Thus, the four identified hub genes involved in the MAPK signaling pathway (i.e., KRAS, JUN, RAP1B, and TNF) may demonstrate clinical utility in the diagnosis and subtyping of IDD, ultimately leading to improved prevention and treatment strategies.

## Author Contributions

**Yong Liu:** data curation, investigation, methodology, writing original draft. **Xueyan Chen:** data curation, investigation, methodology, validation. **Jingwen Chen:** data curation, investigation, methodology, validation. **Chao Song:** data curation, investigation, methodology, validation. **Zhangchao Wei:** investigation, software, validation, visualization, writing review and editing, funding acquisition. **Zongchao Liu:** investigation, software, validation, visualization, writing review and editing, funding acquisition. **Fei Liu:** data curation, investigation, methodology, writing original draft.

## Ethics Statement

The studies involving humans were approved by the Ethics Committee of the Affiliated Traditional Chinese Medicine Hospital of Southwest Medical University (Ethics Approval Number: KY2022062‐FS01). The studies were conducted in accordance with local legislation and institutional requirements. The participants provided their written informed consent to participate in this study.

## Conflicts of Interest

The authors declare no conflicts of interest.

## Supporting information


**Figure S1.** The PPI network.

**Figure S2.** The AUC values of JUN, KRAS, RAP1B, and TNF were 0.728, 0.725, 0.776, and 0.737, respectively.

**Figure S3.** (A–G) Consensus matrices of the four hub MAPK‐related genes for k = 3–9. (H and I) The CDF plot and the delta area of consensus clustering matrix.

**Figure S4.** (A–G) Consensus matrices of the 1916 DEGs for k = 3–9. (H and I) The CDF plot and the delta area of consensus clustering matrix.

**Table S1.** The list of 267 MAPK‐related genes.

**Table S2.** The GO enrichment analysis for 20 differentially expressed MAPK‐related genes.

**Table S3.** The KEGG enrichment analysis for 20 differentially expressed MAPK‐related genes.

**Table S4.** 1916 DEGs.

**Table S5.** The GO enrichment analysis for 1916 DEGs.

**Table S6.** Clinical data.

## Data Availability

The original contributions presented in the study are included in the article (Supporting Information Material); further inquiries can be directed to the corresponding authors.
