# Supplementary figures and images for: The Significance of MAPK Signaling Pathway in the Diagnosis and Subtype Classification of Intervertebral Disc Degeneration
Source: JOR Spine. 2025 Mar 24;8(1):e70060. doi: 10.1002/jsp2.70060 (PMC11932887; doi:10.1002/jsp2.70060)

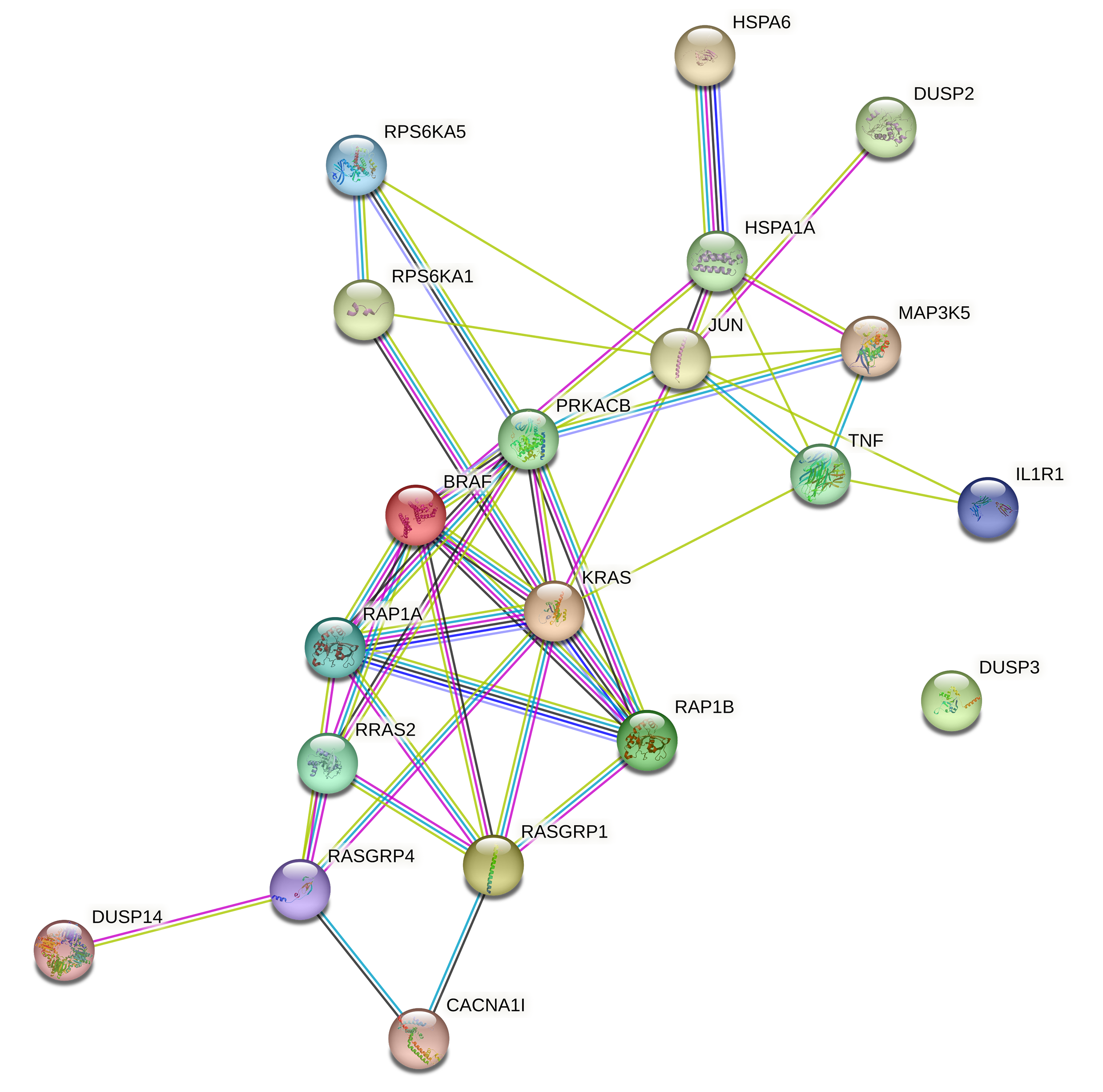

Supplement: Supplementary file 1 — Figure S1. The PPI network. [file JSP2-8-e70060-s005.tif]

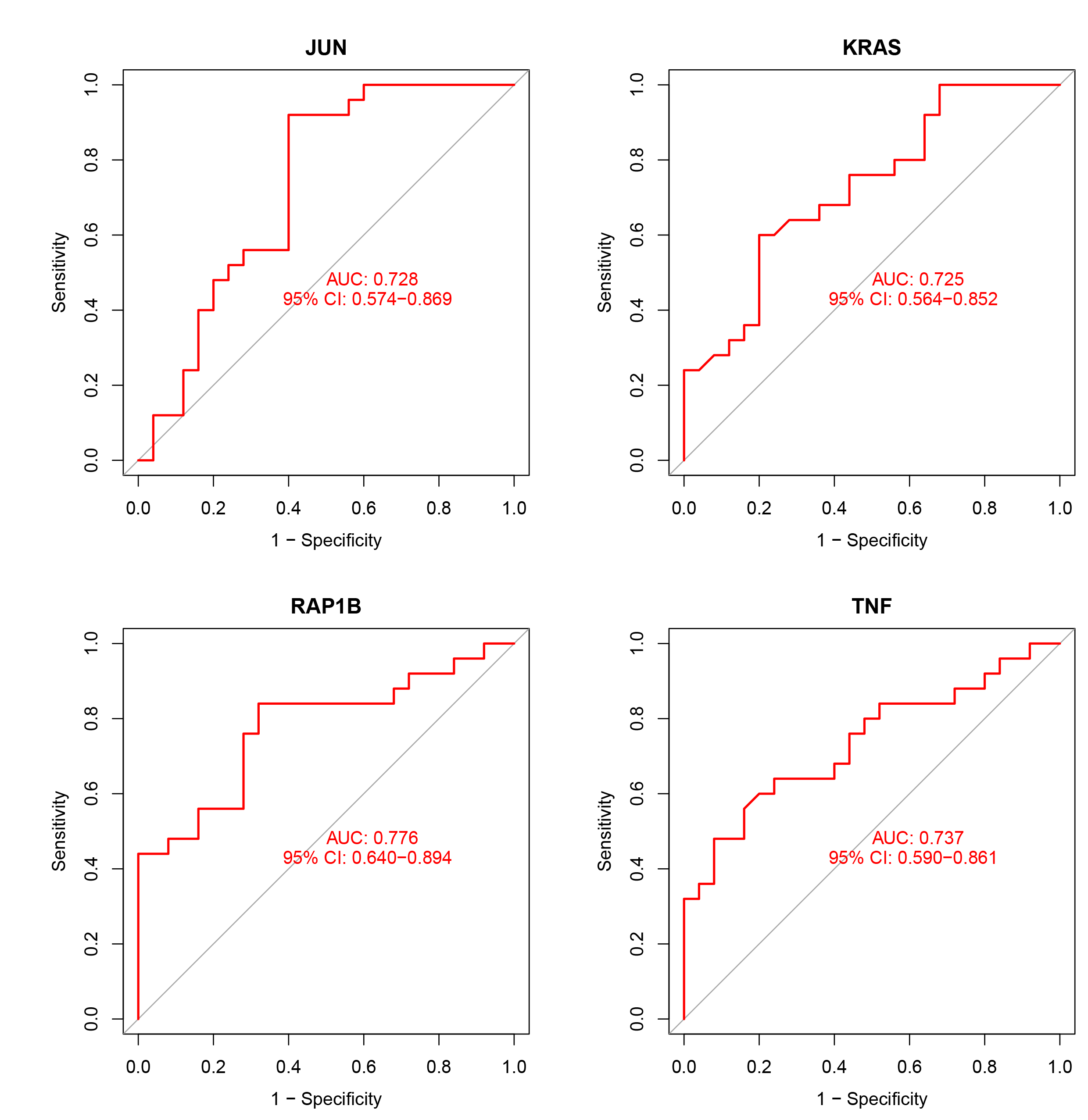

Supplement: Supplementary file 2 — Figure S2. The AUC values of JUN, KRAS, RAP1B, and TNF were 0.728, 0.725, 0.776, and 0.737, respectively. [file JSP2-8-e70060-s007.tif]

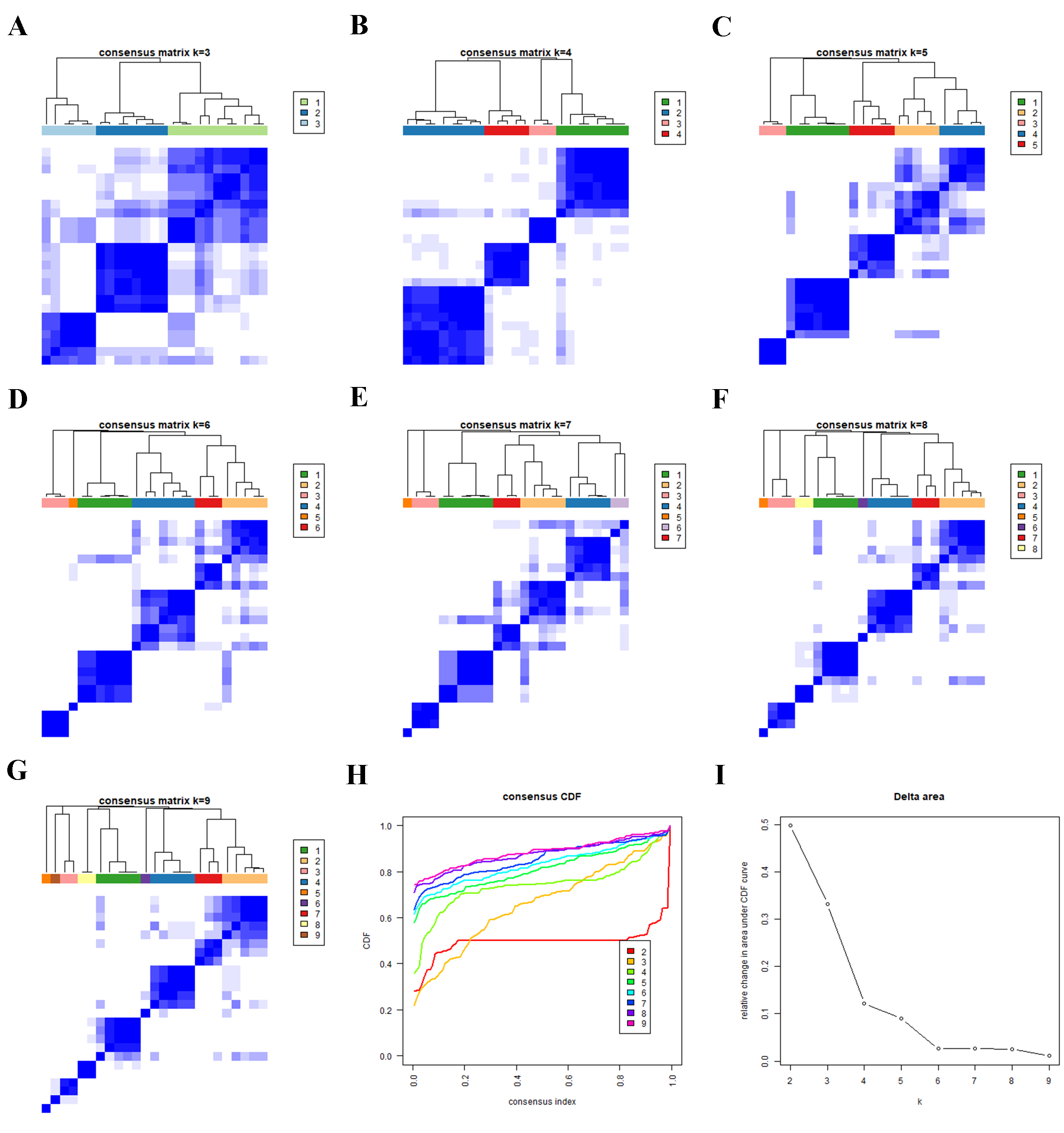

Supplement: Supplementary file 3 — Figure S3. (A–G) Consensus matrices of the four hub MAPK‐related genes for k = 3–9. (H and I) The CDF plot and the delta area of consensus clustering matrix. [file JSP2-8-e70060-s009.tif]

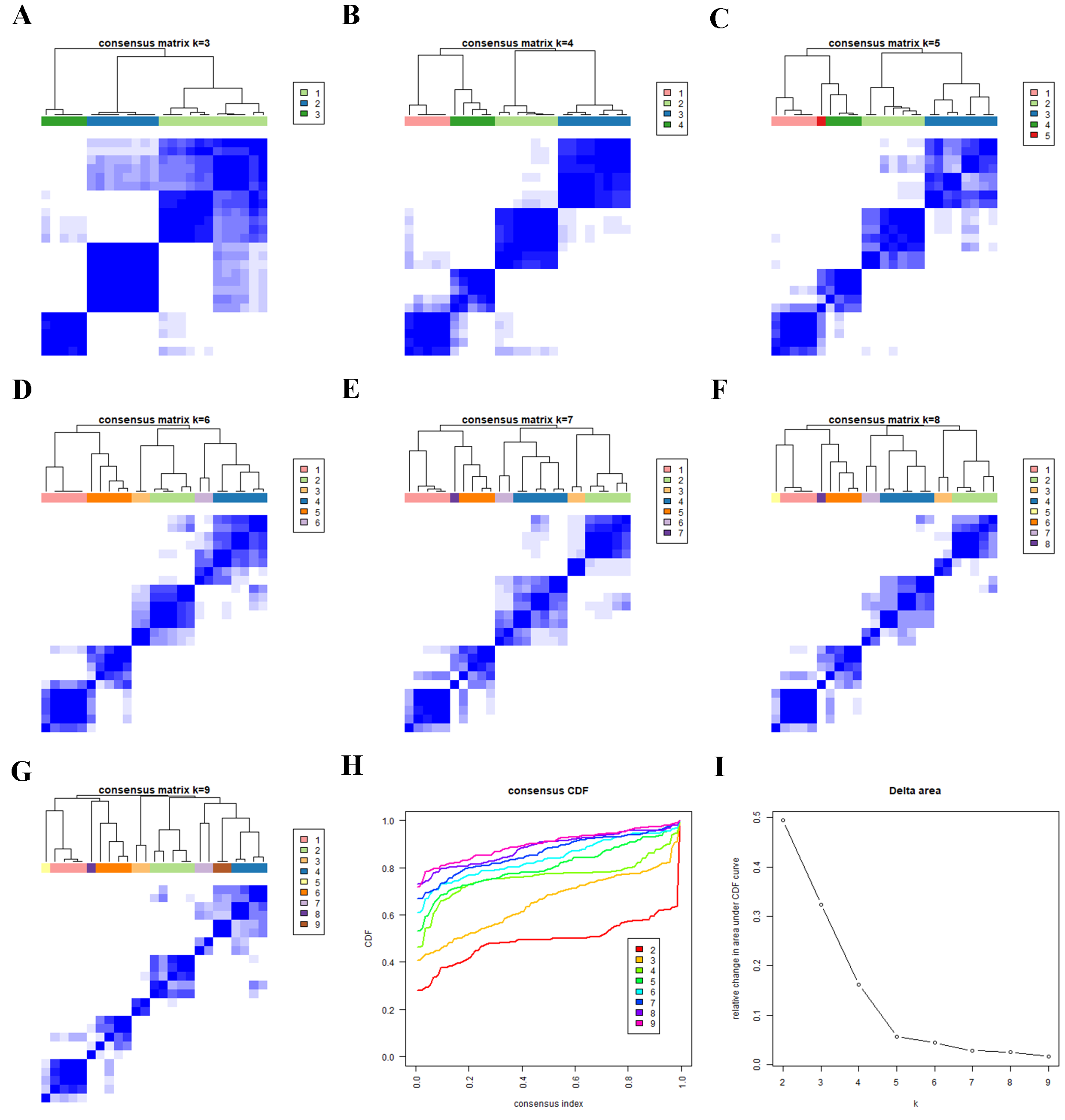

Supplement: Supplementary file 4 — Figure S4. (A–G) Consensus matrices of the 1916 DEGs for k = 3–9. (H and I) The CDF plot and the delta area of consensus clustering matrix. [file JSP2-8-e70060-s001.tif]
